# Assessment of urban river water pollution with urbanization in East Africa

**DOI:** 10.1007/s11356-021-18082-1

**Published:** 2022-01-27

**Authors:** Sophia Shuang Chen, Ismael Aaron Kimirei, Cheng Yu, Qiushi Shen, Qun Gao

**Affiliations:** 1 School of Geographical Sciences, Research Centre of Urban Sustainable Development, Nanjing University of Information Science & Technology, 219 Ningliu Road, Nanjing, 210044 China; 2 State Key Laboratory of Lake Science and Environment, Nanjing Institute of Geography and Limnology, Chinese Academy of Sciences, Nanjing, 210008 People’s Republic of China; 3 Tanzania Fisheries Research Institute, P.O. Box 9750, Dar es Salaam, Tanzania; 4 School of Geography Science and Geomatics Engineering, Su Zhou University of Science and Technology, Suzhou, 215009 China

**Keywords:** Urban river, Gradient analysis, Water pollution, Urbanization, African cities

## Abstract

Anthropogenic pollution plays an important part in deteriorating the water quality of rivers all over the world, especially in urban areas of Africa where water quality monitoring is still seriously constrained by the limited test facility and capability. In this study, for evaluating the impact of urbanization on the river water quality, we investigated four typical urban rivers of Tanzania through the upper-urban-down gradient assessment approach and analyzed by water quality index (WQI) and statistical methods. The physicochemical indices monitored in these rivers revealed that the contents of those indicators of TN, TP, PO_4_^3−^, NH_4_^+^, COD_Mn_, and NO_3_^−^ were accumulated significantly in the lower reaches of the cities, which indicated the life-type pollution characteristics in such urban rivers of Africa. The following main conclusions are achieved from this study. The water quality of 30% of the investigated river sections is in the medium to good status based on the subjective WQI with sensory factors included. Moreover, the sections with obvious water quality decline are mainly limited to the river segments within the urban central area, and severe pollution of water bodies is closely related to large cities, indicating an increasing pollution tendency with the quickly growing population. Therefore, to help formulate water pollution control policies in response to the rapid urban expansion in African countries, it is necessary to adopt an economical and feasible method to carry out early monitoring of surface water quality timely.

**Supplementary Information:**

The online version contains supplementary material available at 10.1007/s11356-021-18082-1.

## Introduction

Water pollution resulting from human activities has become a matter of global concern. The situation has worsened in almost all rivers in Africa, Asia, and Latin America since the 1990s (UNEP, 2016). Deterioration of surface water in less developed countries is caused mainly by urban population–related pollution rather than by agricultural or industrial pollutions (Capps et al. 2016), considering that about 3.2 billion people live in cities, accounting for about 42% of the world’s total population (UN, 2019). Such a large population is still growing rapidly, it is bound to bring about a great pressure on the water quality degradation of rivers, that may endanger the health of residents along the line. Therefore, the assessment of urban river water quality in a wide range of low-income cities plays a key role in the environmental management.

In Sub-Saharan African (SSA) cities, the early study of water quality mainly originates from the attention to urban water supply. Many literatures revealed the eutrophication of Lake Victoria and impacts on the water quality of the around cities such as Jinja, Kampala, and Kisumu (Hecky, 1993; Scheren et al. 2000; Oguttu et al. 2008). Some other researches reported the water pollution in coastal area of Zanzibar resulted from the expanding of population and industrial activities and investigated the baseline concentrations of nutrients (Van Bruggen, 1990), nutrient dynamics and community response to nutrient loading (Johnstonc and Suleiman, 1997), and pesticides and groundwater pollution (Mmochi, 1998; 1997).

The nutrient loads in urban rivers related to some SSA cities have also been explored. For instance, Nhapi and Tirivarombo (2003) investigated the Marimba river through systematical monitoring of water quality and measuring water quantity at different sections and reached that sewage discharges resulted in high residual nutrient levels at a concentration of 13.5 ± 2.0 mg/L in a total of 1842 kg/d for N and a concentration of 2.6 ± 0.6 mg/L in a total of 408 kg/d for P on the entrance into Lake Chivero of Zimbabwe. Some reports assessed nutrient loading of sewage effluent particularly from treatment works in mega cities such as Harare and Durban (Bere, 2007; De Villiers and Malan, 1985). However, in most cases the nutrient loading from urban activity is difficult to accurately measure because of the uncontrolled discharge of sewage. The sewage treatment facilities in mega cities are seriously insufficient and too old, resulting in the untreated direct discharge of a large amount of sewage. For instance, the oxidation ponds built in the late 1950s are still in use in Dar es Salaam, and only about 15% of residents are connected into the sewage network. In Zanzibar, the municipal sewage treatment facilities built in the 1920s are still in operation, with very limited municipal wastewater treated (Mohammed, 2002).Moreover, in the small-middle cities and vast peri-urban areas even there are no sewage pipes and plants, and the wastewater produced there is either discharged in soil via on-site sanitation systems or discharged directly into the rivers and lakes. Several studies have tried to establish the linkage between uncontrolled emissions from informal settlements and nutrient concentrations in rivers (Fatoki et al., 2001; Arimoro et al. 2007), but the nutrient loads can hardly be accurately measured because of the diffused flows and knowledge gaps of nutrient transformation via groundwater (Nyenje et al. 2010). Furthermore, the pit latrines and septic tanks which are used by over 80% residents in SSA cities, often overflow especially during the rains, contaminating surface water or groundwater and increasing health risks in the neighborhoods (Mohammed 2002; Mapunda et al. 2018). Hence, at the current stage sophisticated management strategies based on quantitative nutrient loads may not be the most appropriate pollution control route for SSA cities.

A few studies have reported water quality assessments of the rivers across cities. Beyene et al. (2008) investigated the Borkena river downstream of Dessie and within Kombolcha towns in Ethiopia, analyzed the water quality physicochemical parameters and macroinvertebrate diversity at 10 sampling sites, and distinguished urban affected sites with statistical methods. Moreover, Awoke et al., 2016) disclosed a significant water quality deterioration at those sites affected by urban landscapes as well as agriculture activity, coffee processing based on the investigation of four large rivers, and then put forward practical requirements of environmental management for such specific sites. Some other studies have made a quantified analysis of the contributions from domestic sewage and industrial wastewater on water quality in urban areas (Haddis et al., 2014), compared nutrient concentrations of rivers between urban and rural areas (Yu et al., 2018), or assessed the impacts of different land uses (van der Hoven et al., 2017), for the sake of identifying those areas where immediate resource management strategies were needed. However, in view of the large number of cities and diversified watershed types, the spatial studies accounting for both the land environmental features and water quality parameters are relatively quite limited, and more researches are needed to provide generalized evaluation factors and methods (Hellar-Kihampa, 2017).

As briefly overviewed above, accurate assessment of nutrient loads is seriously constrained by the dispersed discharge of sewage as well as the limited monitoring capacity and technology. There are seldom research reports focusing on finding solutions closely based on local situations for such areas. Therefore, exploring the localization and standardization of water quality monitoring and evaluation methods for urban areas in Africa is urgent to be strengthened. The purpose of this study is to explore a way to rapidly diagnose water quality degradation in the data-deficient areas through a systematic water quality assessment of four urban rivers in Tanzania, aiming at a proactive and sustainable water pollution control in low-income countries.

## Study area

**Fig. 1:**
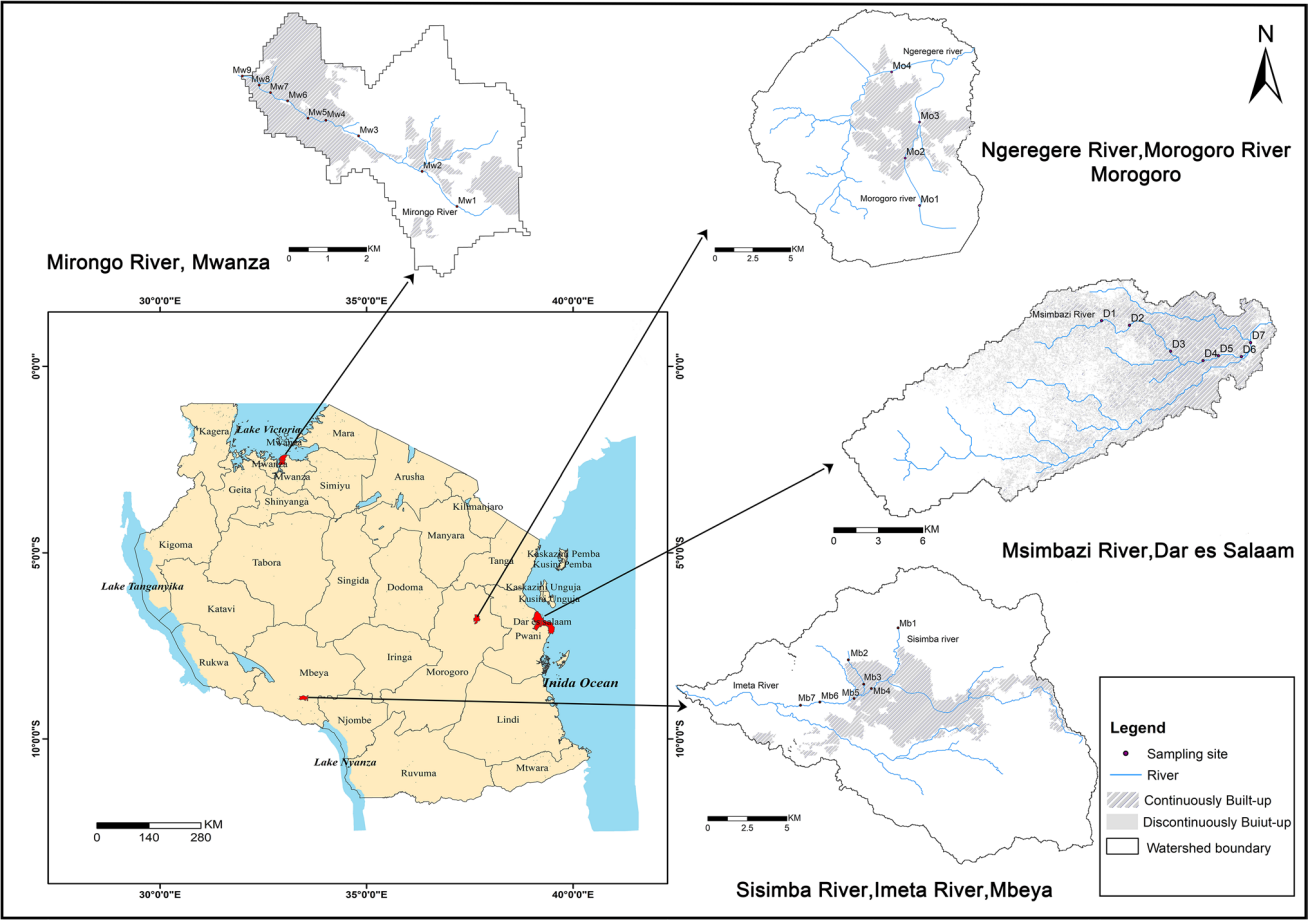
Location of the cities and monitoring sites of Msimbazi river in Dar es Salaam, Mirongo river in Mwanza, Imeta river in Mbeya, and Ngerengere-Morogoro river in Morogoro

Tanzania as one of the Sub-Saharan African countries, had an estimated total population of 59.1 million, with the urban inhabitants accounting for about 33.8% of the total population in 2018. Since the year 2000, it has maintained an average annual population growth rate of 3.0% (UN, 2019). Tanzania belongs to a tropical climate with distinct dry and wet seasons. The weather has some differences along with the change of landforms. The northeast costal areas, Kilimanjaro, and the region around Lake Victoria have the long rainy season usually from March to May and between October and December for the short rainy season (Kimirei et al., 2017). However, the southern, western, and central areas of the country have predominantly one wet season, which covers from October to April. Considering spatial and scale distribution, we selected the urban rivers going through four cities for our study (Fig. 1). The catchment boundary was generated based on SRTM DEM data (https://srtm.csi.cgiar.org), and land uses were obtained based on Google Earth. The land use structure varies by the four investigated catchments. The artificial surface occupies 55.4%, 34.2%, 13.7%, and 14.0%, respectively, of the Msimbazi, Mirongo, Imeta, and Ngerengere-Morogoro watersheds, while the percentages of cultivated land for the corresponding watersheds are 1.1%, 65.7%, 72.1%, and 56.2%. Also, the average population density varies to some extent, which in persons/km^2^ is 14,725, 5188, 3474, and 4185, respectively, for the Msimbazi river basin in Dar es Salaam, Mirongo river basin in Mwanza, Imeta river basin in Mbeya, and Ngerengere-Morogoro river basin in Morogoro based on the census data of 2012 (NBS, 2013). The specific description of each city-river case is given as follows.

### Dar es Salaam city — Msimbazi river

Dar es Salaam is the largest city and an economic center of Tanzania, which has a total area of 1628 km^2^ and a total population of 4,360,000 in 2012 (NBS, 2013). The Msimbazi river runs through this city from west to east. It flows through the suburban areas from upstream to downstream, discontinuous built-up areas, high-density urban areas, and estuary and tidal areas into the Indian Ocean at the Salender Bridge nearby the Palm Beach Hotel. The areas the Msimbazi river flowing through are flat and have rather low water flows during the dry season.

### Mwanza city — Mirongo river

Mwanza is the second largest city of Tanzania, which had a population of 710,000 in 2012 (NBS, 2013). Its municipal districts cover a total area of 1325 km^2^ (including 425 km^2^ land and 900 km^2^ water areas). The Mirongo river runs through its urban areas from southeast to northwest and then flows into Lake Victoria, and Mirongo river is the uppermost river flowing into the lake within the scope of Mwanza city. This channel is parallel to the city’s major external traffic highway B6 and constitutes one of the city’s major extension axes. The upstream section of the Mirongo river originates from a rural area; then it flows through industrial areas, urban areas, and finally into the lake. The areas the Mirongo river is flowing through are flat and have gentle water flow during the dry seasons.

### Mbeya* city — Imeta river*

Mbeya is an important and the fifth largest city in the south of Tanzania. It has a population of about 380,000 (NBS, 2013), and its municipal districts cover a total area of 252.36 km^2^. The Imeta river is the main river passing through the center of Mbeya city. It originates from natural springs in mountainous and forested lands and is the drinking water source for the city of Mbeya. In the middle section, several small tributaries merge into it. The downstream is relatively flat and collects pre-treated wastewater from urban sewage treatment plants. The Imeta river has many other functions, such as floodwater discharge and sewage drainage from unconnected households.

### Morogoro* city — Ngerengere-Morogoro river*

The Morogoro city, locating at approximately 190 km southwest of Dar es Salaam, is an important city in the mid-east section of Tanzania. Its municipal districts cover a total area of 290.02 km^2^ with a population of about 320,000 in 2012 (NBS, 2013). The Ngerengere river is the main river running through its urban areas, with the Mindu Dam constructed in the upstream serving as the city’s main drinking water source. The Ngerengere-Morogoro river, a network of main streams and tributaries, flows through forest lands, urban residential areas, and farmlands. The Morogoro river, the chief tributary in the downstream, passes through dense residential areas.

## Methodology

### Upper-urban-down gradient approach of river water monitoring

Gradient analysis, developed in the context of vegetation survey, has been adopted as a common method to investigate the effects of urbanization on ecosystem properties (Kong and Nakagoshi, 2006), through which the plant factors are observed by setting continuous land sample site on the urban–rural gradient. Given the substantially reported consistent urbanization syndrome of rivers such as a loss of sensitive taxa and increased pollutant loads accompanied with urban development (Parr et al., 2016), in this study we take river as the urban–rural gradient zone, and water samples are collected along the river to assess the impact of urbanization on water quality parameters. The proposed approach is called upper-urban-down gradient method in combination with the rural–urban gradient of the change from river upstream to downstream. The specific river segmentation method employed is described as follows.

Firstly, the site locating in the upper reaches of the river, mostly surrounded by forests before entering the city, is often chosen as a reference point to compare the impact of urban or agricultural activity on the river. Secondly, the river segment within the urban area is regarded as an experimental site. The change of water quality compared with the reference point should be able to indicate the influence of urban activity. Thirdly, the lower reach of the river far after passing through the city is also adopted as an experimental site. The changes compared with the upstream reference point and with the urban point can be used to determine the self-purification ability of the river to a certain extent. Also, the water quality at this point can largely indicate the potential impact of the city to the external aquatic environment.

In dividing the upper, middle/urban, and lower reaches of the river, defining the middle segment is the key to segmentation, which is determined by urban boundaries. But in the SSA cities, extensive informal settlements make the border between urban and rural areas blurred. Due to the large portion of urban population scattered in the peri-urban areas without urban infrastructure, it is not appropriate to delineate urban areas according to the built-up areas, which might be too small. For instance, the residents living in the unplanned informal settlements occupy about 70% of its population in Dar es Salaam and 21% in Mwanza (Hambati and Yengoh, 2018). If all the informal settlements are included, it is some difficult to draw a clear line between these discontinuous urban settlements and rural settlements. In order to distinguish the urban from the countryside reasonably, we first identify the artificial surface in the investigated watersheds, and then determine the urban area according to the population density. Since the density varies greatly within a watershed, such as the Msimbazi basin, where the population density varies from 50 persons/km^2^ to 46,738 persons/km^2^ by wards, some population density threshold can be chosen to divide urban and rural areas. A relative value, such as the median value of the urban population density of the investigated watershed, is adopted as the criterion to distinguish between urban and rural wards.

With this approach, a total of 27 study sites have been set up for the four selected rivers. From upstream to downstream, the first site is located at the trunk’s headwater, while the last one at the river mouth into the lake (for Mirongo river) or into the ocean (for Msimbazi river), or outside of the continuous urban settlements (for Imeta river, and for Ngerengere-Morogoro river). The middle sites are distributed by equal distance in line with landform characteristics, land use types, and concurrently considering tributary confluence points and pollution discharge points (Fig. 1). The sampling site numbers are arranged in ascending order from upstream to downstream. At last, all the determined monitoring sites have been classified into upper-urban-down three types as listed in Table 1.

**Table 1:** Gradient distribution of sampling sites along the four urban rivers

	Headwater/rural/ peri-urban	Urban	Urban downstream
Msimbazi R	D1, D2	D3, D4, D5, D6	D7
Mirongo R	Mw1*, Mw2, Mw3	Mw4, Mw5, Mw6, Mw7, Mw8	Mw9
Sisimba-Imeta R	Mb1, Mb2	Mb3, Mb4, Mb5	Mb6, Mb7*
Ngerengere-Morogoro R	Mo1	Mo2, Mo3, Mo4	/

*An industrial park locating at the upstream of Mw1; a sewage treatment plant discharging between Mb6 and Mb7.

### Sampling descriptions, water quality parameters, and their measurements

The sampling was performed in March 2016 (wet season) and August 2016 (dry season) with an interval of 5 months. Two parallel samples were taken for chemical analysis, and each collected water sample was further split into two portions: one was filtered through a 0.45-mm glass fiber filtration membrane and then kept in a pre-labelled 60-mL polypropylene bottle; the other unfiltered sample was kept in a pre-labelled 60-mL polypropylene bottle for analyzing TN and TP. All samples were kept at or below 4 °C pending analysis.

A multi-parameter water quality instrument (YSI ProDSS, Xylem Brand) was used to carry out in-situ measurement of physical parameters, including dissolved oxygen (DO), oxidation reduction potential (ORP), pH, electrical conductivity (EC), temperature, total dissolved solids (TDS), and turbidity. The chemical parameters, including total nitrogen (TN), total phosphorus (TP), ammonia nitrogen (NH_4_^+^-N), nitrate nitrogen (NO_3_^−^-N), nitrite nitrogen (NO_2_^−^-N), soluble reactive phosphorus (SRP), calcium (Ca), magnesium (Mg), sulfate (SO_4_^2−^), chlorine (Cl^−^), fluorine (F^−^), and permanganate index (COD_Mn_), were analyzed according to standard methods (APHA, 1998). The potassium persulfate oxidation-UV spectrophotometer method and a UV–Vis spectrometer (UV-2450PC, Shimadzu) were used to determine TN and TP. Other chemicals and nutrients were determined with the Flow Continuous Chemistry Analyzer (San +  + , Skalar) at the State Key Laboratory of Lake Science and Environment (Nanjing, China).

### Data analysis and WQI calculation

Firstly, the water quality indices measured in this study are plotted to illustrate their variation by seasons and sites. Then the values of each physicochemical parameter are grouped by river segments according to the upper-urban-down gradient approach, and one-way ANOVA is used to examine the differences between the urban impaired and reference sites (headwater/peri-urban site) using SPSS version 11.0 (IBM Inc, USA). If the difference is statistically significant, it is considered that the water quality in the urban and downstream sites has been degraded. Moreover, the critical levels of involved parameters were collected and used as reference values for relative comparison and judgment for the measured indices (as summarized in Table 2), according to standard of the Tanzania Bureau of Standards (TZS) for drinking water, or referenced indicators as recommended by WHO (World Health Organization), US-EPA (Environmental Protection Agency of United States), EU-WFD (Water Framework Directive of European Union), and China-MEP (Ministry of Environmental Protection of China) for surface water. If the value is within the limits, the water quality is considered to be healthy.

**Table 2:** Statistical summary of the physical and chemical parameters of the investigated river water during wet and dry seasons

		Wet season (March)				Dry season (August)			
Parameters	Critical level	Minimum	Maximum	Mean	SD(*n* = 27)	Minimum	Maximum	Mean	SD(*n* = 27)
TN (mg/L)	< 1.0^b^	0.55	45.1	7.52	9.65	0.80	60.10	8.96	13.92
TP (mg/L)	< 0.1^a^	0.02	2.66	0.44	0.76	0.02	2.62	0.51	0.80
NO_2_^−^(mg/L)		0.00	0.27	0.11	0.09	0.01	0.11	0.04	0.03
NH_4_^+^(mg/L)	< 0.3^a^	0.04	17.98	1.73	4.01	0.07	14.58	2.63	4.18
NO_3_^−^(mg/L)	< 10^b^	0.00	7.12	2.33	2.12	0.07	5.88	2.72	1.87
PO_4_^3−^(μg/L)	< 70^a^	0.00	1368	190	332	2.32	845.95	133.39	209.64
F^−^(mg/L)		0.06	12.98	0.87	2.45	0.13	11.14	2.05	2.51
Cl^−^(mg/L)		0.69	711.51	167.24	261.15	0.56	867.10	169.87	240.99
SO_4_^2−^(mg/L)		0.44	376.93	51.52	91.68	0.81	150.05	37.43	43.34
DO (mg/L)	> 7^a^	2.20	17.85	9.89	4.25	0.47	14.53	7.21	3.45
ORP		− 10.00	355.00	204.1	98.24	86.00	497.00	282.63	115.54
TDS (mg/L)		30	6270	819	1340	67	15,200	1062	2876
EC (μS/cm)	< 980^d^	47.00	10,310	1467	2575	104	23,100	1643	4372
Turbidity (NTU)	< 5/25^b^	9.69	252.00	79.63	65.76	4.79	318.00	46.47	76.77
Temperature (°C)		18.93	32.31	25.75	3.70	12.25	30.04	23.97	4.33
pH		-	-	-	-	6.38	12.14	7.48	1.28
*COD*_Mn_ (mg/L)	< 6^c^	-	-	-	-	4.91	282.46	37.72	83.01
Ca(mg/L)		2.24	295.18	44.13	-	-	-	-	-
Mg(mg/L)		0.15	833.41	42.96	-	-	-	-	-

^a^The limit levels recommended by US-EPA and EU, citied in Awoke et al., 2016

^b^,National Environmental Standards Compendium of Tanzania, TZS 789:2003-Drinking (potable) water—Specification.

cEnvironmental Quality Standards for Surface Water of China (GB3838-2002) Class III.

^d^UNEP 2016. A Snapshot of the World’s Water Quality: Towards a global assessment. United Nations Environment Programme, Nairobi, Kenya. Appendix C2 Case study 5 – Vaal.

In addition, water quality index (WQI), a general index integrated multiple parameter is used to quantitatively interpret the water quality of the investigated urban rivers, which is easily compared between different water bodies and seasons, and thus water quality level can be effectively understood by managers and decision-makers in the water sector through simply observing individual values and corresponding scales (Chang et al., 2001; Akkoyunlu and Akiner, 2012). The NSF-WQI, originally developed by National Sanitation Foundation of the USA, is modified to keep in accordance with the available measured parameters in this study, and the calculation equation is shown as below:

$$WQI=k\frac{\sum \limits_{i=1}^{n}{C}_{i}{P}_{i}}{\sum \limits_{i=1}^{n}{P}_{i}}$$

where, *k* is a positive constant of no greater than 1, with 1 indicating non-polluted water body and 0.25 indicating black and smelly water body. And *n* denotes number of water quality index parameter, *C*_i_ denotes the standardized value of index *i* value; *P*_*i*_ denotes the relative weight of water quality index parameter *i*, with the value ranging from 1 to 4, where *P*_*i*_ = 1 stands for the least important and 4 for the most important parameter. The values of *C*_i_ and concentrations retrieved from Conesa curves (Conesa, 1995), as cited by Pesce and Wunderlin (2000) and Akkoyunlu and Akiner (2012), are provided in Table S1, and the base for determining the value of *k* in this study is provided in Table S2. Since *k* represents the visual and odour score through subjective judgment, the WQI value with *k* included is specifically called subjective WQI and denoted as WQI_sub_, and the WQI without considering *k* is denoted as WQI_obj_, further comparisons between the two kind of WQI values are discussed in the later parts.

The WQI value is in the range of 0 ~ 100, and the water quality levels of the evaluated water bodies are divided into different categories according to the value range (Jonnalagadda and Mhere, 2001; Sehnaz Sener et al., 2017). Whereby, if the WQI value is located within 0–25, it indicates that the water quality of that particular water body is in the category “extremely poor”, and the WQI value within 25–50 stands for the “poor” category, then within 50–70 for the “medium” category, 70–90 for the “good” condition, and within 90–100 for the “excellent” category water body.

## Results and analysis

### Water quality physicochemical features of the investigated rivers in dry and wet seasons

The results of the physical and chemical parameters are summarized in Table 2 and presented in Appendix Figs. S1–S6. Based on *t*-test of the 27 paired samples, the parameter values between dry and wet seasons do not show statistically significant difference reaching the level of *p* < 0.05 except for few parameters such as temperature, DO, ORP, turbidity, nitrite (NO_2_^−^), and ammonium (NH_4_^+^). The temperature values in wet period are a bit higher than those recorded in dry period, and turbidity values are relatively higher during the wet season than those in the dry season resulted from the increased sediment entering the water body with the flood during the rainy season. The mean value of DO concentration was measured to be 9.89 mg/L (*SD* = 4.25) in wet period and 7.21 mg/L (*SD* = 3.25) in dry period (Table 1). While the mean value of NH_4_^+^ in the wet was measured to be 1.73 mg/L (*SD* = 4.01), lower than 2.63 mg/L (*SD* = 4.18) obtained in the dry season. The higher DO level in wet period than that in dry season was in agreement with the monitoring data of the Pangani river basin of Tanzania (PBWO/IUCN, 2007). It seems difficult to give a very reasonable explanation for the seasonal differences, Hellar-Kihampa et al. (2013) ascribed the lowered ionic concentrations and the higher DO value to the dilution effect during rainfall, and the relatively higher concentrations of oxygen demanding substances including NH_4_^+^ during the dry season may mainly account for the seasonal differences of DO distribution in the studied rivers.

As compared to the adopted critical levels listed in Table 2, the percentages meeting the requirements of the observed values for TN, TP, NH_4_^+^, orthophosphate (PO_4_^3−^), and COD_Mn_ were counted to be 13%, 41%, 50%, 50%, and 27%, respectively. The turbidity index performed poorly, over 98% of the observations were unqualified by lower than 5 NTU critical level, and still 59% were substandard even by the relatively broad criteria below 25 NTU. However, 100% of the measured values for NO_3_^−^ met the critical level of less than 10 mg/L, and the parameter DO performed better with 57% of the DO observations higher than the critical level of 7 mg/L.

Among the investigated four rivers, the water quality parameters of Msimbazi river of Dar es Salaam have appeared relatively higher values at all sites. As shown in Appendix Fig. S3, although the values of F^−^ were all in low concentrations and did not show significant differences between different rivers, the values of Cl^−^ and SO_4_^2−^ were significantly higher in the Msimbazi river. Also, the Msimbazi river had relatively higher TN (Appendix Fig. S4) and TP values among all the investigated rivers, and the highest TP values were recorded in sites D3 and D4 during the wet and dry seasons, respectively (Appendix Fig. S5). Similarly, phosphate concentrations showed the same trend as that of TP. The concentration of permanganate index (*COD*_Mn_) was higher in Msimbazi river and the highest value was found at the site D4 (Appendix Fig. S6). This results revealed that the Msimbazi river suffered severe organic pollution.

### Comparison of pollutant concentrations at variant upper-urban-down sites compared to the reference site

**Fig. 2:**
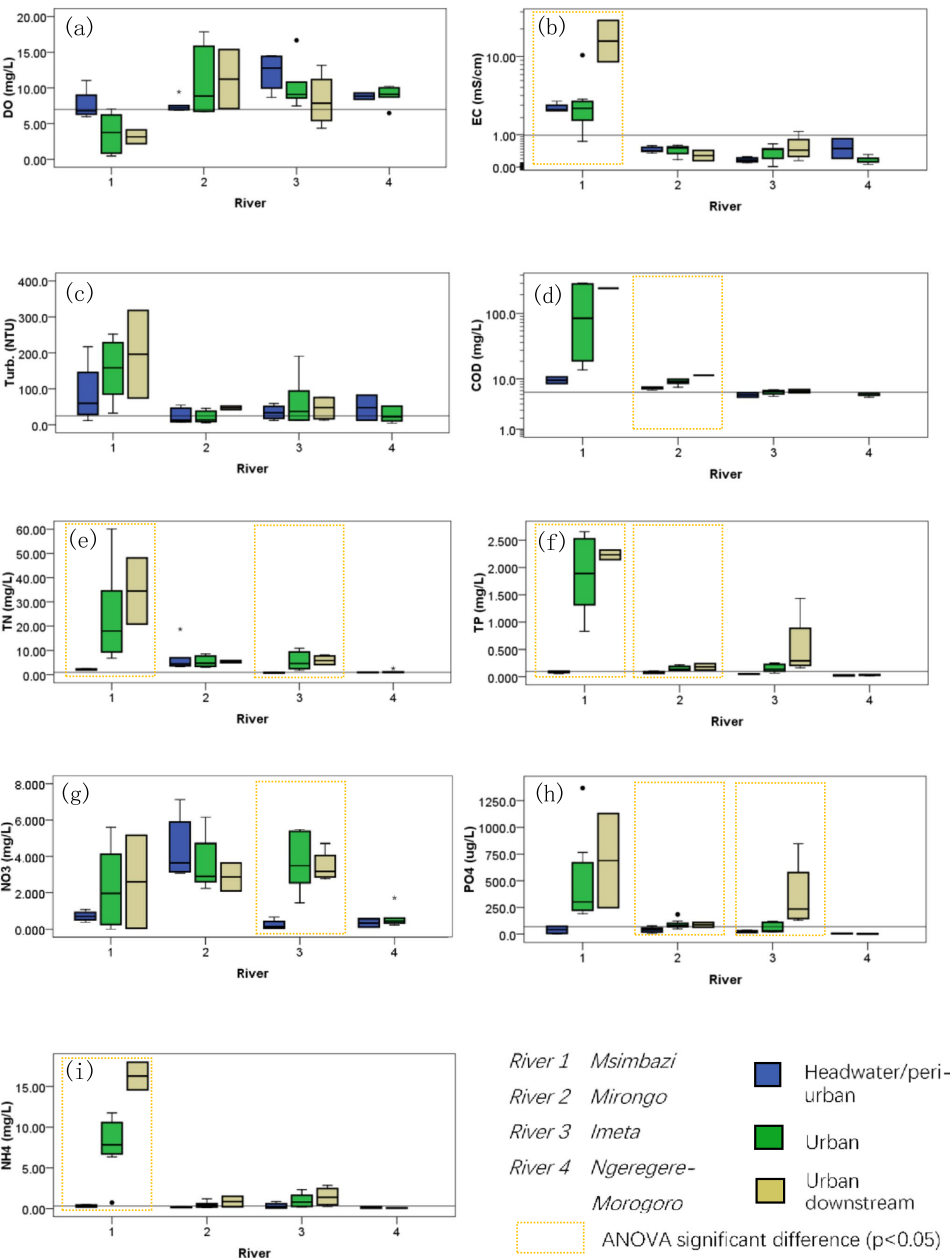
Comparison and statistical analysis of water quality parameter values in box plots with reference value at upper-urban-down gradient segments of the investigated four urban rivers

The isndex values showed spatial variation by the different upper-urban-down sites in addition to the differences between rivers, as shown in Fig. 2. The box plots of 9 physicochemical parameters reflected the values and some statistical analyses via one-way ANOVA at headwater/peri-urban, urban, and urban downstream segments of the investigated rivers.

The concentrations of nutrients and *COD*_Mn_ were observed to increase in the order of headwater/peri-urban site < urban site < downstream site for all rivers, with the exception of NO_3_^−^ in No. 2 Mirongo river of Mwanza (Fig. 2g). The concentrations of TP, PO_4_^3−^, and NH_4_^+^ in the headwater/peri-urban sites were lower than the recommended allowable threshold values, indicating a relatively healthy state, while the concentrations in the urban area and downstream are comparatively higher. Moreover, the one-way ANOVA statistical analysis of TP, PO_4_^3−^, and NH_4_^+^ showed significant differences with *p* < 0.05 for different segments of Msimbazi river, and the differences of COD_Mn_, TP, and PO_4_^3−^ were significant between various segments of Mirongo river, also significant differences of TN and PO_4_^3−^ for Imeta river.

The DO values recorded were relatively high compared with the recommended minimum allowable values except for No. 1 Msimbazi river and were lower in the urban sites than those from the non-urban control sites in general. However, there was no simple and consistent trend appeared for the four rivers. The measured values of turbidity were mostly beyond the critical allowable values for all the investigated rivers with those from No.1 Msimbazi river exceptionally high.

### WQI-based assessment for water quality degradation diagnosis

**Fig.3:**
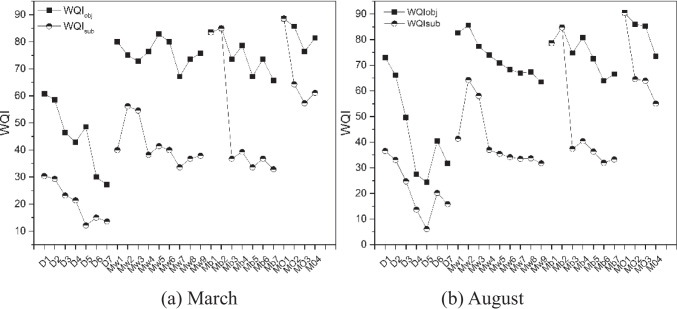
Spatial variation of *WQI*_obj_ and *WQI*_sub_ in March wet period and August dry season

Spatial variations of integrated water quality as presented by *WQI*_obj_ and *WQI*_sub_ are shown in Fig. 3. The values of *WQI*_obj_ were in general higher than those of *WQI*_sub_. For all the 27 selected sites, most of the sites were in medium (*WQI* = 50–70) and good (*WQI* = 70–90) status based on *WQI*_obj_, only 19% of the sites in the wet period and 15% in dry season were in poor (*WQI* = 25–50) status. However, the water quality was significantly worse when considering the water body’s sensory state such as color and smell. Based on *WQI*_sub_ with the inclusion of *k* values, only 30% of the sites were in medium and good status in both the wet and dry seasons, more than half in poor status.

Specifically, according to the value of *WQI*_sub_ in both wet and dry seasons, 64% of the sites in Msimbazi river were in extremely poor status and 36% of the other sites in poor status, the worst site was D5 which originated from the direct influence of a local printing and dyeing mill. From the upstream to downstream, the water quality became worse, indicating that the Msimbazi river was severely polluted by associated urban development (Appendix Fig. S7).

For Mirongo river, 78% of the sites were in poor status both in wet period and in dry season, 22% of the other sites were in medium status, with the worst site Mw7 locating in the densely populated area. The similar trend exhibited that the water quality became worse from the upstream to the downstream, the exceptional site was Mw1 with a *WQI*_sub_ (41.9 in dry season, 40.0 in wet season) lower than its downstream point Mw2 (64.6 in dry season, 56.2 in wet season), which was ascribed to its locating near an industrial area (Appendix Fig. S8).

For Imeta/Sisimba river, 71% of the sites were in poor water quality status and the other 29% sites in good status. Those sites with good water quality were located in the woodland or areas with less disturbances from human activities. The worst site was Mb7 (with *WQI*_sub_ 32.8) during the wet season and Mb6 (30.8) during the dry season. The sites Mb7 and Mb6 were ascribed to be influenced by the local wastewater plant and associated urban development, respectively (Appendix Fig. S9).

For Ngerengere river, 75% of the sites belonged to the medium status during both wet and dry season. Moreover, 25% of the other sites exhibited good water quality status during the wet and during the dry season, the water quality of the other 25% sites was excellent. The highest *WQI*_sub_ value of 91.7 was recorded at the site of MO1 in August, which served as the drinking water source for the Morogoro City. The other sites were located in the city and obviously influenced by the urban development (Appendix Fig. S10).

For the four investigated rivers, both *WQI*_obj_ and *WQI*_sub_ showed an apparent decreasing trend in general from the headwater to the downstream sites. The *WQI*_obj_ values of headwater/peri-urban sites were close to each other in either dry or wet season, around 80 except for the Msimbazi river. Table 3 shows the diagnosis of water quality degradation in the four rivers based on the summarized *WQI* values for various sites in the segments of headwater/peri-urban, urban, and urban downstream of each river.

**Table 3:** Water quality status of various sites in headwater/peri-urban, urban, and urban downstream segments for the investigated four urban rivers

		Water quality category			Level change after urban areas	
		Headwater/peri-urban	Urban	Urban downstream	Dry season	Wet season
1	Msimbazi river	M-G	EP-M	P	-2	-1
2	Mirongo river	G	M-G	M-G	-1	0
3	Sisimba/Imeta river	G	M-G	M-G	-1	-1
4	Ngerengere-Morogoro river	G-VG	M-G	/	/	/

The water quality of the urban and urban downstream sites shows poor or medium to good, while those in the headwater and upper reaches are medium to good or good to very good, which clearly indicates that the rivers are obviously influenced by the associated cities. Such urban-related pollution is more pronounced in the dry season, so that the downstream water quality of all rivers has dropped by 1–2 grades. During the wet season, municipal sewage is diluted to some extent, and the urban downstream water quality of only two rivers (Msimbazi and Sisimba) drops by one grade. Therefore, the degree of river influenced by urban discharge can be determined by the decline of water quality in the upper and lower reaches of the river, which should be helpful to provide the basis for environmental policy making.

## Discussion

Since many of the Sub-Saharan African countries are largely in the early stages of industrialization, there are much less industrial pollutants compared with the developed nations/regions. This situation is reflected in the organic pollution with mainly domestic pollution sources of the investigated urban river waters. The anthropogenic ammonium sources increasing with the growing population have led to a high concentration of nitrogen in the form of ammonium in the river through the direct discharge of wastewater or via runoff in this kind of low-income cities (Tromboni and Dodds, 2017; Moncayo-Estrada et al. 2017). The phosphorus emissions from detergents and household food consumption constituted primary sources of phosphorus pollution in water (Xiong et al. 2020). The results of this study are essentially in consistency with the previous reports. Moreover, it is interesting and unsurprising to find that the contents of those indicators of TN, TP, PO_4_^3−^, NH_4_^+^, COD_Mn_, and NO_3_^−^ are accumulated significantly in the lower reaches of the city, which indicates the impacts of life-type pollution and the shortage of sewage system coverage in densely populated catchments (Pacheco and Sanches Fernandes, 2016).

In this study, we focus more on the analysis and discussion of the spatial characteristics of the population-related pollution as briefly summarized below. Firstly, among the 27 section sites surveyed in this study, 80% of the sections were in good to middle level based on the *WQI*_obj_ values without considering the sensory factor. However, if judged from *WQI*_sub_ with sensory indicators taken into account, the water quality evaluation declined by 1–2 grades, then the section numbers reaching the good to middle level reduced to about 30%. Since the sensory indicators are mainly judged based on some visual or olfactory subjective factors, such as water color, odor, and suspended solids, thus *WQI*_sub_ values decrease significantly for most urban river waters, which have been polluted with surficial floating trashes and/or terrible smells, because the garbage is usually dumped along the rivers, and in some areas domestic sewage is directly discharged into the river as well. The actual pollution reflected by the objective index *WQI*_obj_ values is not as serious as the subjective feeling. Therefore, at this stage, the pollution, which mainly originates from daily life, is relatively easy to mitigate or eliminate as long as domestic pollution sources can be controlled in time and strengthen water treatment measures, while it is suggested to be put into action immediately.

Secondly, the results show that the section with obvious water quality decline is mainly limited to the river segments within the urban central area. Generally, the urban section water quality exhibited inferior indices compared to the peri-urban section for all the four rivers investigated, the results of statistical analysis of nitrogen and phosphorus nutrient indices also corroborated the significant difference of water quality between the two different river segments. Lack of infrastructure and high concentration of large population with lots of domestic pollutants directly discharging into the rivers caused degradation of water quality in urban river sections, which were also revealed in many previous reports (Haddis et al 2014; van der Hoven et al. 2017; Yu et al. 2018). Moreover, the water pollution in the central urban area also influenced the downstream water quality, which led to the poor water quality indices of the downstream sections. Such performance was different from the situation reported in some literature, where the downstream water quality was generally restored after flowing through a longer distance (Beyene et al. 2008). Some cities selected in this study, such as Dar es Salaam and Mwanza, are quite close to the ocean or lake, and the persistent pollution loads from urban settlements to the river tend to exceed the rivers’ self-purification capacity within such a short distance between the urban central area and the downstream entrance). Thus, the shorter distance to the urban section of the downstream sampling sites in this study together with the weak self-purification ability of the rivers may account for the results. Most of the investigated rivers have no buffers and are mostly bare with little vegetation in the banks, thus lacking sufficient ability to filter and purify the water. The self-purification ability of these rivers deserves enough attention and needs further investigation for effectively guiding the management of urban river water (Chinyama et al. 2016).

Thirdly, the serious pollution of water bodies related to large cities indicates an increasing pollution trend with increasing population (Liyanage and Yamada, 2017). Comparing the results of the four urban rivers, it was shown that overall the water quality of the Msimbazi river crossing the Dar es Salaam city was poorer, where on average the *WQI* was in the medium level in the upstream and dropped into poor level for the middle and downstream sections. Highly concentrated urban activities led to an obvious decline of the water quality in urban river sections, the agglomeration of large population in urban fringe areas (Juma et al. 2014) and the concentration of agricultural activities serving the city in the surrounding areas significantly degraded the overall water quality of the whole river. For the other three cities in the survey, the water quality showed a relatively gentle gradual decline tendency with about one grade reduced in the urban section relative to the upstream, and the average *WQI* still reached good to middle level (Fig. 3, Table 3). It demonstrated that the water quality decline was inconsistent with the spatial distribution of pollution sources and did not spread broadly along river systems or with hydrological processes. Nevertheless, the difficulty and challenge of management will increase with the rapid increase in the large city numbers in African countries and the quick growth of the total population and urban residents.

The limitation of this paper is that mainly the physical and chemical indices are employed without the biological indices included, thus the water quality assessment parameter types can be more comprehensive with the biological factor included. Indicators such as *Escherichia coli* and benthic invertebrates will be considered in subsequent studies to comprehensively reflect the ecosystem health and water quality (Arimoro et al., 2015; Mwaijengo et al., 2020). Furthermore, although *WQI*-integrated assessment method is widely adopted in the water quality assessment research literature around the world, the samples to determine the parameter standard values are mainly from the massive water quality data of domestic rivers in the USA. (Samantray et al., 2009; Bonanno and Giudice, 2010; Akkoyunlu and Akiner, 2012). The suitability of these standard values to Africa rivers needs further study. For example, the *DO* indicator is the most commonly used physicochemical index, while the current used *DO* standard values cannot accurately reflect the actual water quality of the investigated areas. The measured *DO* values were around 7 mg/L even for some sites of poor water quality, which belongs to a good level as compared to the reference values adopted during *WQI* evaluation (Table S1), thus it is urgent to develop a much elaborate and localized standard and determine suitable *DO* limits matching the local environmental situation. In this regard, this study also contributes to the accumulation of water quality data to address the serious deficiencies in local standard threshold studies.

In the future, much more cross-basin water quality researches are encouraged to support environmental management pertinent to different watersheds (Neal et al. 2011; Roy et al. 2016). Limited by economic and technical conditions, spatial monitoring and assessment of water quality are difficult to carry out in the vast urban and rural areas of SSA countries. This study has revealed the effectiveness of the upstream-urban-downstream gradient evaluation method for the investigated four cities in Tanzania, which can be applied to the rapid diagnosis of water quality problems in more similar cities.

## Conclusions

Anthropogenic pollution is making the water quality of rivers and streams all over the world worse, such a situation is further exacerbated by the high-intensity land use and inadequate environmental management. The gradient assessment approach of three-segment monitoring proposed in this study can deal with the limitations of economic and technical conditions in low-income cities and obtain reliable data for scientific evaluation of water pollution. Specifically, this study determined the overall pollution level, the urban influence degree, and the key pollution river section with possible reasons analyzed and some measures suggested, which should contribute to the development of urban pollution control policies more in line with the actual situations in Tanzanian cities.

In conclusion, based on the study on four typical African urban rivers, it is disclosed that the water quality is in good to medium grade for 80% of the investigated river sections based on the objective *WQI*, while with the sensory indicators included, the water quality generally declines by 1–2 grades, with medium to good sections only reaching 30%. The sections showing obvious water quality decline are mainly limited to the river segments of the urban central area. Highly concentrated urban activities in the large city, together with the agglomeration of large population in urban fringe areas, and the concentration of agricultural activities, lead to the obvious decline of the water quality in urban river sections associated with mega cities.

Therefore, it is demonstrated that the water quality decline is consistent with the spatial distribution of pollution sources and does not spread broadly along river systems or with hydrological processes. With the rapid increase of large cities in African countries and the quick growth of urban population, the difficulty and challenge of pollution control will definitely increase. Thus, it is necessary to adopt an economical and feasible method to carry out early monitoring of surface water quality in order to help formulate water pollution control policies timely in response to the rapid urban expansion.

## Supplementary Information

Below is the link to the electronic supplementary material.

Supplementary file1 (DOCX 1013 KB)

## Data Availability

The datasets used and/or analyzed during the current study are available from the corresponding author on reasonable request.

## Abbreviations

*VG*: Very good with *WQI*_obj_ > 90
*G*: Good with *WQI*_obj_ between 70 and 90
*M*: Medium with *WQI*_obj_ between 50 and 70
*P*: Poor with *WQI*_obj_ between 25 and 50
*EP*: Extremely poor with *WQI*_obj_ < 25
