# Supplementary material for: Assessment of urban river water pollution with urbanization in East Africa
Source: Environ Sci Pollut Res Int. 2022 Jan 27;29(27):40812–25. doi: 10.1007/s11356-021-18082-1 (PMC9135893; doi:10.1007/s11356-021-18082-1)
Supplement: Supplementary file 1 — Supplementary file1 (DOCX 1013 KB) [file 11356_2021_18082_MOESM1_ESM.docx]

**Supplementary Materials**

Appendix

**Table S1 Parameters adopted for WQI calculation**

| Water quality indices | Unit | *P*_i_ | Centralized indexes (*C*_i_) | | | | | | | | | | |
| --- | --- | --- | --- | --- | --- | --- | --- | --- | --- | --- | --- | --- | --- |
|  |  |  | 100 | 90 | 80 | 70 | 60 | 50 | 40 | 30 | 20 | 10 | 0 |
| T | ℃ | 1 | 21/16 | 22/15 | 24/14 | 26/12 | 28/10 | 30/5 | 32/0 | 36/-2 | 40/-4 | 45/-6 | >45/-6 |
| pH |  | 1 | 6.9–7.5 | 6.7–7.8 | 6.5–8.3 | 6.2–8.7 | 5.8–9.0 | 5.5–9.5 | 5.0–10.0 | 4.5–10.5 | 4.0–11.5 | <4.0; | >11.5 |
| DO | mg/L | 4 | ≥7.5 | >7 | >6.5 | >6 | >5 | >4 | >3.5 | >3 | >2 | ≥1 | <1 |
| EC | μS/cm | 1 | <750 | <1000 | <1250 | <1500 | <2000 | <2500 | <3000 | <5000 | <8000 | ≤12000 | >12000 |
| Turbidity | NTU | 2 | <5 | <10 | <15 | <20 | <25 | <30 | <40 | <60 | <80 | ≤100 | >100 |
| COD | mg/L | 3 | *<*1.2 | *<*5.0 | *<*7.5 | *<*10.0 | *<*12.5 | *<*15.0 | *<*20.0 | *<*25.0 | *<*30.0 | ≤40.0 | *>*40.0 |
| NH_4_^+^ | mg/L | 3 | <0.01 | <0.05 | <0.1 | <0.2 | <0.3 | <0.4 | <0.5 | <0.75 | <1 | ≤1.25 | >1.25 |
| NO_3_^-^ | mg/L | 2 | <0.5 | <2 | <4 | <6 | <8 | <10 | <15 | <20 | <50 | ≤100 | >100 |
| PO_4_^3-^ | mg/L | 1 | <0.025 | <0.05 | <0.1 | <0.2 | <0.3 | <0.5 | <0.75 | <1.0 | <1.5 | ≤2 | >2 |

**Table S2 Basis for determining *k* value**

| *k* value | Basis descriptions |
| --- | --- |
| 1 | Water without apparent contamination (clear or with natural suspended solids) |
| 0.75 | Slight contaminated water (apparently), indicated by slight non-natural color, foam, slight turbidity due to natural reasons |
| 0.5 | Contaminated water (apparently), indicated by non-natural color, slight to moderate odor, high turbidity (no natural, suspended organic solids, etc.) |
| 0.25 | Highly contaminated water (apparently), indicated by blackish color, hard odor, visible fermentation, etc. |







(a) March (b) August

Fig.S1 Spatial variation of some physical parameters in March and August







(a) March (b) August

Fig.S2 Spatial variation of electricity conductivity (EC) in March and August


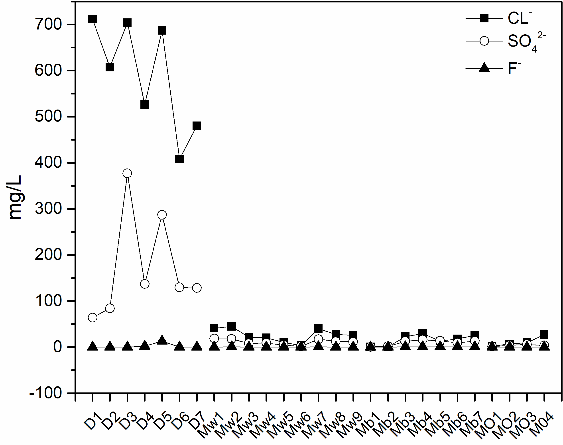




(a) March (b) August

Fig.S3 Spatial variation of chloride, fluorine and sulfate in March and August







(a) March (b) August

Fig.S4 Spatial variation of inorganic nitrogen and total nitrogen in March and August







(a) March (b) August

Fig.S5 Spatial variation of orthophosphate and total phosphorus in March and August





Fig.S6 Spatial variation of permanganate index in August







(a) March (b) August

Fig.S7 Spatial variation of WQI_obj_ and WQI_sub_ in March and August for Msimbazi river







(a) March (b) August

Fig.S8 Spatial variation of WQI_obj_ and WQI_sub_ in March and August for Mirongo river







(a) March (b) August

Fig.S9 Spatial variation of WQI_obj_ and WQI_sub_ in March and August for Imeta/Sisimba river







(a) March (b) August

Fig. S10 Spatial variation of WQI_obj_ and WQI_sub_ in March and August for Ngeregere river
